# Simultaneous estimation of global background synaptic inhibition and excitation from membrane potential fluctuations in layer III neurons of the rat entorhinal cortex *in vitro*

**DOI:** 10.1016/j.neuroscience.2007.05.016

**Published:** 2007-07-29

**Authors:** S.D. Greenhill, R.S.G. Jones

**Affiliations:** Department of Pharmacy and Pharmacology, University of Bath, Claverton Down, Bath, BA2 7AY, UK

**Keywords:** entorhinal cortex, synaptic noise, background excitation, background inhibition, voltage fluctuations, neuronal excitability, ACSF, artificial cerebrospinal fluid, E_Bg_, background excitation, EC, entorhinal cortex, GYKI 53655, 1-(4-aminophenyl)-3-methylcarbamoyl-4-methyl-3,4-dihydro-7,8-methylenedioxy-5H-2,3-benzodiazepine, I_Bg_, background inhibition, I:E, ratio of inhibitory to excitatory conductance, NBQX, 6-nitro-7-sulfamoylbenzo[f]quinoxalone-2,3-dione disodium, NMDA, *N*-methyl-d-aspartate, sEPSC, spontaneous excitatory postsynaptic current, sIPSC, spontaneous inhibitory postsynaptic current, TTX, tetrodotoxin, UBP-302, (S)-1-(2-amino-2-carboxyethyl)-3-2-(carboxybenzyl)pyrimidine 2,4-dione, V_m_D, measurement of fluctuations in membrane potential, 2-AP5, 2-amino-5-phosphonopentanoic acid

## Abstract

It is becoming clear that the detection and integration of synaptic input and its conversion into an output signal in cortical neurons are strongly influenced by background synaptic activity or “noise.” The majority of this noise results from the spontaneous release of synaptic transmitters, interacting with ligand-gated ion channels in the postsynaptic neuron [Berretta N, Jones RSG (1996); A comparison of spontaneous synaptic EPSCs in layer V and layer II neurones in the rat entorhinal cortex *in vitro*. J Neurophysiol 76:1089–1110; Jones RSG, Woodhall GL (2005) Background synaptic activity in rat entorhinal cortical neurons: differential control of transmitter release by presynaptic receptors. J Physiol 562:107–120; LoTurco JJ, Mody I, Kriegstein AR (1990) Differential activation of glutamate receptors by spontaneously released transmitter in slices of neocortex. Neurosci Lett 114:265–271; Otis TS, Staley KJ, Mody I (1991) Perpetual inhibitory activity in mammalian brain slices generated by spontaneous GABA release. Brain Res 545:142–150; Ropert N, Miles R, Korn H (1990) Characteristics of miniature inhibitory postsynaptic currents in CA1 pyramidal neurones of rat hippocampus. J Physiol 428:707–722; Salin PA, Prince DA (1996) Spontaneous GABA_A_ receptor-mediated inhibitory currents in adult rat somatosensory cortex. J Neurophysiol 75:1573–1588; Staley KJ (1999) Quantal GABA release: noise or not? Nat Neurosci 2:494–495; Woodhall GL, Bailey SJ, Thompson SE, Evans DIP, Stacey AE, Jones RSG (2005) Fundamental differences in spontaneous synaptic inhibition between deep and superficial layers of the rat entorhinal cortex. Hippocampus 15:232–245]. The function of synaptic noise has been the subject of debate for some years, but there is increasing evidence that it modifies or controls neuronal excitability and, thus, the integrative properties of cortical neurons. In the present study we have investigated a novel approach [Rudolph M, Piwkowska Z, Badoual M, Bal T, Destexhe A (2004) A method to estimate synaptic conductances from membrane potential fluctuations. J Neurophysiol 91:2884–2896] to simultaneously quantify synaptic inhibitory and excitatory synaptic noise, together with postsynaptic excitability, in rat entorhinal cortical neurons *in vitro*. The results suggest that this is a viable and useful approach to the study of the function of synaptic noise in cortical networks.

Cortical neurons are embedded in a dense, complex network and are the target of tens of thousands of individual synapses. Both excitatory (glutamate) and inhibitory (GABA) synapses continuously release transmitter as a result of action potentials within network interconnections, but also in the form of mono-quantal, activity-independent release (miniature events). The combination of action potential-dependent and -independent release, *in vivo* at least, is thought to aid signal detection through stochastic resonance, where sub-threshold synaptic events are pushed above threshold due to the presence of background activity. Thus, the relative level of inhibitory and excitatory background activity is a reflection of network activity and is instrumental in determining the excitability of any given neuron (Pare et al., 1998; Stevens and Zador 1998; Destexhe and Pare, 1999; Ho and Destexhe, 2000; Rudolph and Destexhe, 2001; Stacey and Durand, 2000; Fellous et al., 2003; Shu et al., 2003a).

We have been studying spontaneous release of both glutamate and GABA onto neurons in the entorhinal cortex (EC) using whole cell patch clamp recording of spontaneous synaptic currents *in vitro* (see Jones and Woodhall, 2005). However, this approach does not lend itself well to relating the level of background activity to cellular excitability. Generally, experimental recording conditions are established to record either excitatory or inhibitory currents in isolation. The somatic recording location means that more distally located currents may not be detectable. Most importantly, the inclusion of blockers of voltage-gated ion channels in the patch pipette solution, to improve space clamp etc., largely precludes meaningful estimates of cellular excitability. Sharp-electrode intracellular recording allows for the latter, but does not provide high enough electrical resolution for direct observation of small amplitude background synaptic events. Rudolph et al. (2004) have recently proposed a method of estimating global background synaptic conductances from measurement of fluctuations in membrane potential (termed V_m_D) derived from sharp electrode intracellular recordings (see also Korngreen, 2004). The analytic expression of mean and standard deviation of membrane potential distribution permits simultaneous estimation of the global background excitation mediated by glutamate acting at AMPA receptors (E_Bg_) and background inhibition (I_Bg_, due to GABA acting via GABA_A_ receptors). Importantly, the use of intracellular recording allows us to simultaneously obtain measurements of cellular excitability and, thus, to relate excitability to relative levels and changes in E_Bg_ and I_Bg_.

The approach of Rudolph et al. (2004) has essentially been applied to high conductance states characteristic of cortical networks *in vivo*. In the current experiments we have asked whether we can meaningfully estimate E_Bg_ and I_Bg_ under the quiescent conditions recorded under baseline conditions in EC slices *in vitro*. We have then used a variety of pharmacological manipulations to examine to what extent activity dependent release contributes to background activity, whether the measured conductances truly reflect activation of glutamate and GABA receptors, and whether there are interdependent network relationships discernible from alteration of either conductance. Finally we have determined whether changes in background conductances result in altered cellular excitability.

## Experimental procedures

All experiments were performed in accordance with the U.K. Animals (Scientific Procedures) Act 1986, European Communities Council Directive 1986 (86/609/EEC) and the University of Bath ethical review document. Every attempt was made to keep the number of animals used to a minimum and also to minimize any suffering and stress inflicted. EC slices were prepared from male Wistar rats (60–70 g) as described previously (Jones and Heinemann, 1988). Following decapitation the brain was rapidly removed and immersed in chilled (4 °C) oxygenated artificial cerebrospinal fluid (ACSF). Slices (400 μm) were cut with a Vibroslice (Campden Instruments, Loughborough, UK) and transferred to ACSF bubbled with 95% O_2_/5% CO_2_ at room temperature. After recovery of at least 1 h, individual slices were transferred to an interface chamber perfused with oxygenated ACSF (1.5 ml/min) maintained at 32±0.5 °C for recording. ACSF consisted of (in mM): NaCl (126), KCl (3.75), MgSO_4_ (1.5), NaHCO_3_ (19), NaH_2_PO_4_ (1.4), CaCl_2_, (2) and d-glucose (10), pH 7.4 at recording temperature.

**Fig. 1:**
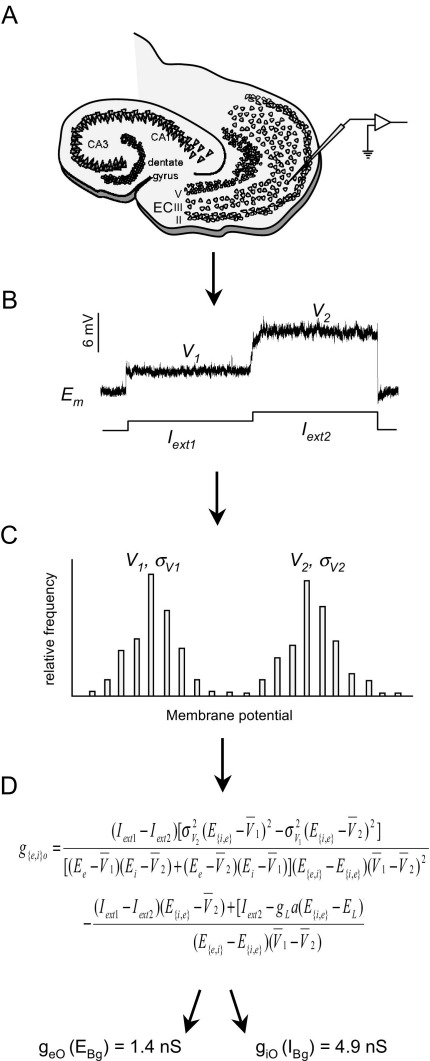
(A) Intracellular recordings were made from layer III neurons in slices of rat EC. (B) At intervals, the membrane potential was depolarized by injection of two known external currents I_ext1_ and I_ext2_. (C) Membrane potential fluctuations at each current level were fitted to Gaussian distributions and the mean and standard deviation determined. In the neuron shown in B, the resting potential was −72 mV. With I_ext1_ at 0.15 nA the Gaussian fit gave V_1_ as −68.7 mV with σ_V1_ at 0.6 mV. At I_ext2_ (0.33 nA), the corresponding values were −60.3 and 1.22 mV. (D) These values, together with the previously determined reversal potentials for AMPA (E_e_) and GABA_A_ (E_i_) (see text), and the leak conductance (g_L_a) obtained during each recording (see text), were used to calculate I_Bg_ and E_Bg_ using the V_m_D method described by Rudolph et al. (2004).

Intracellular voltage recordings were made with sharp electrodes filled with potassium acetate (3 M) from pyramidal neurons in layer III of the medial EC using an Axoprobe 1A amplifier (Axon Instruments, Foster City, CA, USA) in bridge mode (Fig. 1A). Data were acquired using a Digidata 1200 and Axoscope software (Axon Instruments). Typically (*n*=6), these neurons had a resting potential of −72.2±0.5 mV, input resistance of 79±6 mΩ and action potential amplitude (from threshold) and half-widths of 72.1±1.6 mV and 0.32±0.01 ms, respectively. In an initial series of experiments synaptic responses were evoked via a bipolar stimulating electrode placed in the lateral EC. In six neurons EPSPs mediated by AMPA receptors were isolated by blockade of *N*-methyl-d-aspartate (NMDA), GABA_A_ and GABA_B_ receptors using appropriate antagonists (see below). The mean reversal potential of AMPA receptor mediated responses (+6.6±2.0 mV) was determined by recording EPSPs at different membrane potentials. Similar experiments (*n*=6) were performed, substituting an AMPA antagonist for the GABA_A_-antagonist, in the blocking cocktail, to determine the reversal potential of GABA_A_-receptor mediated IPSPs (−66.7±1.0 mV).

At intervals during subsequent experiments to estimate E_Bg_ and I_Bg_, neurons were depolarized (for 15–20 s) to two sub-threshold levels by injection of known positive currents (I_ext1_ and I_ext2_) via the recording electrode (Fig. 1B). The values of the currents differed from neuron to neuron, but were maintained the same throughout any individual experiment. I_ext2_ was chosen to elicit a depolarization to within 1–2 mV of action potential threshold and I_ext1_ was adjusted to depolarize the neuron to about halfway between I_ext2_ and resting membrane potential. Membrane potential fluctuations at these two levels were fitted to Gaussian distributions and the mean and variance of the membrane potential determined (Fig. 1C). Leak conductance in each neuron was calculated from the ohmic response produced by a small (0.1 nA 100 ms) hyperpolarizing current, injected at resting membrane potential. These parameters, together with the mean reversal potentials derived from preliminary experiments, allowed us to apply the V_m_D equation (Fig. 1C; equation 11 in Rudolph et al., 2004) to quantify background inhibitory and excitatory conductances resulting from global network input onto individual neurons.

Excitability of neurons was tested with depolarizing current pulses during intervals between conductance estimate protocols. Short (50 ms) pulses were injected with incrementally increasing amplitudes (0.1–1.5 nA) to determine the threshold for eliciting a single action potential. Longer (250 ms) supra-threshold pulses were used to elicit a train of spikes, to monitor firing frequency.

The following drugs were used: 6-nitro-7-sulfamoylbenzo[f]quinoxalone-2,3-dione disodium (NBQX) (AMPA/kainate receptor antagonist, Tocris, Bristol, UK), 1-(4-aminophenyl)-3-methylcarbamoyl-4-methyl-3,4-dihydro-7,8-methylenedioxy-5H-2,3-benzodiazepine (GYKI 53655) (AMPA receptor antagonist, gift from Dr. Dick Evans, Bristol University), 2-amino-5-phosphonopentanoic acid (2-AP5) (NMDA receptor antagonist, Tocris), (S)-1-(2-amino-2-carboxyethyl)-3-2-(carboxybenzyl)pyrimidine 2,4-dione (UBP-302) (GluR5-containing kainate receptor antagonist, gift from Dr. David Jane, Bristol University), bicuculline methochloride (GABA_A_ receptor antagonist, Tocris), tetrodotoxin (TTX) (Alamone Laboratories, Jerusalem, Israel).

Gaussian fits of membrane potential and statistical analysis (paired *t*-tests) were done with GraphPad Prism software (GraphPad, San Diego, CA, USA). All values are expressed as mean±S.E.M.

## Results

Currently, the V_m_D method is applicable to GABA_A_- and AMPA-mediated conductances, and does not take into account GABA_B_- or NMDA receptor–mediated activity. However, it is unlikely that spontaneous activation of GABA_B_-receptors occurs in EC neurons (Woodhall et al., 2005), and although spontaneous currents mediated by postsynaptic NMDA receptors do occur in slices, such events are very infrequent (Berretta and Jones, 1996a). Thus, the vast majority of background synaptic noise in EC neurons is mediated via AMPA and GABA_A_ receptors.

These experiments were conducted on a total of 43 layer III neurons in the medial EC. Mean E_Bg_ in these neurons was 2.8±0.4 nS and I_Bg_ was 13.3±2.3 nS. There was considerable variability in the two conductances from neuron to neuron. There are a number of practical reasons why this may be so, including the day to day variation in quality of slices, whether slices were selected from more dorsal or ventral regions, variations in preservation of intrinsic connectivity etc. It seems unlikely that the variations may be due to sampling of different neuronal types. We studied regular spiking neurons in layer III, and these have a homogenous pyramidal cell morphology and electrophysiological characteristics (e.g. Dickson et al., 1997; Tahvildari and Alonso, 2005). Despite the neuron-to-neuron variation I_Bg_ was consistently greater than E_Bg_. The ratio of inhibitory to excitatory conductances (I:E) was clearly in favor of inhibition, and this is similar to the situation estimated from naturally occurring up states of activity recorded in ferret occipital cortex *in vitro* (Rudolph et al., 2004), and cat neocortex *in vivo* (Rudolph et al., 2007). In other experiments in this laboratory we have been studying spontaneous excitatory postsynaptic currents (sEPSCs) and spontaneous inhibitory postsynaptic currents (sIPSCs) using whole cell patch clamp recording in layer III neurons. While it is difficult to directly relate global conductances to spontaneous currents, it is pertinent that in a sample of seven neurons sIPSCs had a mean frequency and amplitude of 12.4±2.9 Hz and 37.4±3.2 pA, while in a further seven neurons the corresponding values for sEPSCs were 6.6±1.2 Hz and 11.5±0.9 pA (S. E. L. Chamberlain and R. S. G. Jones, unpublished observations).

### Effect of blocking activity dependent release

**Fig. 2:**
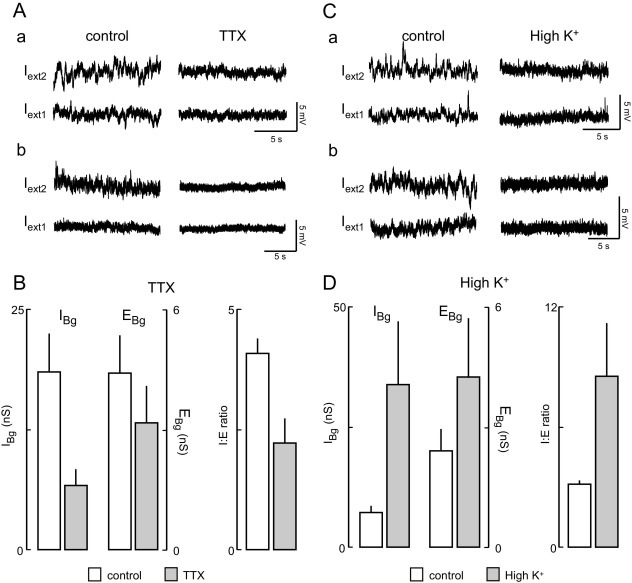
Effects of blocking activity dependent release. (A) The traces are examples of intracellular recordings of membrane potential taken from two individual neurons (Aa, Ab) at the two levels of injected current used for calculation of E_Bg_ and I_Bg_ as described in Fig. 1. Note the increased voltage fluctuations at the more depolarized levels (I_ext2_) in these and the examples shown in all subsequent figures. Addition of TTX resulted in a dampening of spontaneous activity. (B) The bars show the mean values of I_Bg_ and E_Bg_ in the absence (clear bars) and presence of TTX. I_Bg_ was reduced to a greater extent than E_Bg_ with a consequent decrease in I:E in favor of excitation. (C) Examples of voltage recordings from two neurons (Ca, Cb). With increased [K^+^]_o_ spontaneous activity was increased resulting in more high frequency activity and a thickening of the membrane voltage traces. (D) Summary data for elevated [K^+^]_o_ in all neurons show an in increase in both I_Bg_ and E_Bg_.

Blockade of voltage-gated sodium channels would remove background release driven by spontaneous firing in both principal and interneurons but leave miniature events intact. TTX (1 μM, *n*=6) significantly reduced both I_Bg_ (from 18.3±3.8 to 6.7±1.5 nS, *P*<0.01) and E_Bg_ (4.4±1.0 to 3.6±1.1 nS, *P*<0.01). Thus, a much greater proportion of the background GABA release appears to depend on spontaneous firing in inhibitory neurons, compared with activity dependent release of glutamate. These results are summarized in Fig. 2A and B. Again, it is relevant that whole cell voltage clamp recordings in our laboratory show that sIPSC frequency in layer III neurons is reduced by around 50–60% by TTX whereas the toxin only elicits a fall of around 20% in sEPSCs (S. E. L. Chamberlain and R. S. G. Jones, unpublished observations).

### Effect of increasing overall network excitability

In seven neurons we elevated [K^+^]_o_ from 3.75 mM to 7.5 mM to induce a generalized increase in network activity and to increase the release of both glutamate and GABA (Fig. 2C). This manipulation elevated both E_Bg_ and I_Bg_. E_Bg_ was elevated from 2.4±0.5 nS to 4.3±1.3 nS (*P*=0.04). I_Bg_ was even more profoundly increased, rising nearly fivefold from 7.1±1.6 nS to 34.0±12.7 nS (*P*=0.03). The I:E increased further in favor of inhibition, from 3.1±0.2 to 8.6±2.6 (*P*>0.05, Fig. 2D). This could be because the inhibitory neurons are already closer to firing threshold than principal cells, and the depolarizing effect of the high K^+^ drives them at a disproportionately high rate. Such an effect could also be compounded by an increase in glutamate-mediated excitatory drive onto the interneurons.

The increase in [K^+^]_o_ depolarized membrane potential by 2.9±0.7 mV. It would also be expected to directly affect spike threshold per se, so it is difficult to draw any conclusions with respect to excitability changes and changes in background synaptic activity. Firing threshold was substantially reduced (from 20.0±0.6 to 13.1±0.3 mV. However, surprisingly, there was no change in the number of spikes evoked by a supra-threshold depolarizing pulse (4.8±0.3 to 4.8±0.9). It is possible that the increased K^+^ enabled easier generation of action potentials, but that this was counteracted by the relative increase in I_Bg_, which then acted to reduce sustained firing.

### Blockade of AMPA receptors

**Fig. 3:**
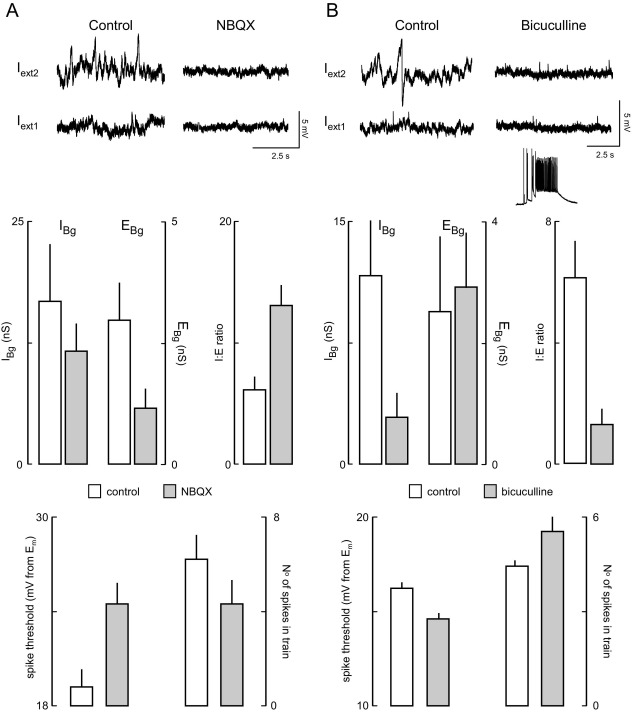
A. The traces in the upper panel are examples of voltage recordings in an individual neuron and the bar graphs in the middle panel are summary data of changes in background synaptic activity following blockade of AMPA-receptors with NBQX. The antagonist reduced E_Bg_ as expected, but also concurrently reduced I_Bg_. The overall effect was to increase the I:E in favor of inhibition, with a concurrent increase in spike threshold and decrease in repetitive firing (lower panel bar graphs). (B) GABA_A_-receptor blockade dramatically decreased I_Bg_, with little change in E_Bg_. The resulting decrease in ratio in favor of excitation was accompanied by an overall increase in excitability. The inset trace below the voltage records in the upper panel show a spontaneous epileptiform discharge recorded in the illustrated neuron shortly after the estimate of I_Bg_ and E_Bg_ revealed that the ratio of inhibition to excitation in this neuron had fallen to around 1:1.

The effect of the AMPA-receptor antagonist, NBQX (10 μM, *n*=11) is summarized in Fig. 3A. As expected, the drug decreased E_Bg_ from 2.9±0.7 to 1.2±0.5 nS (*P*=0.02). Surprisingly, however, there was a concurrent reduction in I_Bg_ (from 16.9±5.8 to 11.6±2.8 nS; *P*>0.05). The most likely explanation for the latter observation is a decreased AMPA-receptor-mediated excitatory drive onto the GABAergic neurons, resulting in a decreased rate of spontaneous firing of the interneurons. This conclusion was supported by experiments where NBQX was perfused in slices pre-treated with TTX (*n*=3). In these neurons E_Bg_ decreased from 3.6±1.1 to 1.4±0.5 nS). Concurrently, there was little change in I_Bg_ (6.7±1.5 to 5.9±2.8 nS). In a further three neurons we have tested the effect of the non-competitive antagonist, GYKI 53655 (25 μM), which is more specific for AMPA over kainate receptors. Although control levels were much lower in this small sample, results were similar to NBQX with a reduction in E_Bg_ from 1.4±0.1 to 0.4±2 nS, and in I_Bg_ to 2.0±0.7 from 5.5±0.6 nS.

When AMPA receptors were blocked with NBQX, the I:E increased from 6.2±1.0 to 13.1±1.8 (*P*=0.02, Fig. 3A), further tilting the balance of background synaptic activity in favor of inhibition. This was reflected by an overall decrease in excitability in the same cells. NBQX increased the spike threshold from 19.2±1.0 mV to 24.5±1.3 mV (*P*=0.03) from resting potential while the mean number of spikes generated during a 250 ms supra-threshold depolarizing pulse decreased from 6.2±2.1 to 4.2±1.0 (*P*>0.05, Fig. 3A). Thus, while we cannot say whether the change in excitability arises as a direct consequence of the decreased E_Bg_, it certainly seems to parallel the overall change in I:E. It should be noted that NBQX had no effect on resting potential.

### Blockade of GABA_A_-receptors

The quantification of I_Bg_ using the V_m_D approach relates only to that mediated by GABA_A_-receptors (Rudolph et al., 2004). Addition of the competitive GABA_A_-receptor antagonist, bicuculline (10 μM, *n*=6), caused a profound reduction in I_Bg_ (from 11.5±3.4 to 2.9±1.7 nS, *P*=0.04, Fig. 3B). Concurrently, there was no change in E_Bg_ (2.5±1.2 nS versus 2.9±0.9 nS), suggesting that the loss of network inhibition does not result in increased spontaneous activity in recurrent excitatory connections between principal cells. Continued administration of bicuculline ultimately results in spontaneous epileptiform discharges in most slices (see Jones, 1988). The estimations of background activity here were taken immediately after the first spontaneous discharge (mean time from start of bicuculline 7.4±0.7 min). After regular seizures start it is difficult to obtain the stable baseline membrane potential essential for consistent estimations of background conductances, so it is likely that I_Bg_ may fall further during continued perfusion of bicuculline, and also conceivable that this would lead to changes in E_Bg_ as extensive disinhibition occurred.

Overall, I:E decreased in favor of excitation, from 6.1±1.2 to 1.3±0.5 at the time when spontaneous seizures were initiated. At the same time there was no change in resting potential, but there was an increase in cellular excitability (Fig. 3B), with spike threshold falling from 16.3±0.3 to 14.7±0.3 mV (*P*>0.05) and the number of spikes per train rising from 4.5±0.2 to 5.6±0.4 (*P*>0.05).

In a small group of neurons (*n*=3), we have tested the effect of bicuculline (10 μM) in the presence of TTX. Again, the antagonist had little effect on E_Bg_ (3.6±1.1 nS versus 3.5±1.2 nS), but strongly decreased I_Bg_ (6.7±1.5 to 2.3±1.0 nS), with a change in I:E in favor of excitation (2.2±0.5 to 0.6±0.1).

### Blockade of NMDA receptors

**Fig. 4:**
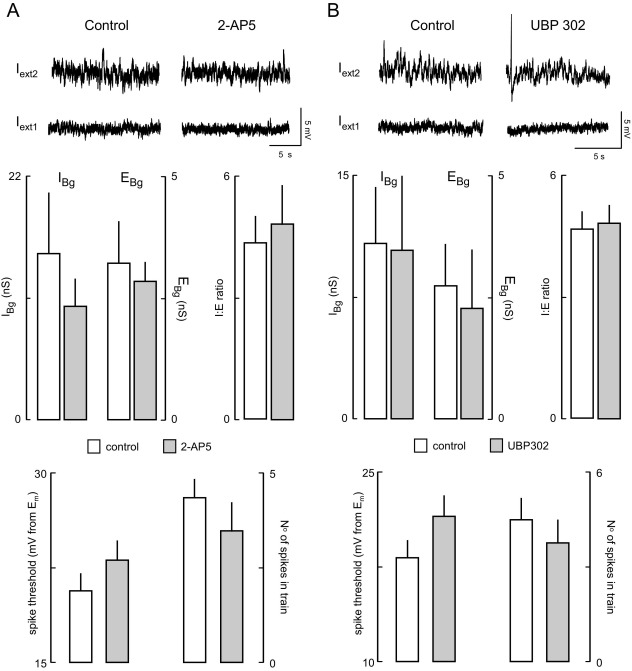
(A) NMDA-receptor blockade slightly reduced I_Bg_ with little overall effect on E_Bg_ and no change in ratio. Excitability was also little altered. (B) Blocking GluR5 kainate receptors had little effect on background synaptic activity or excitability.

Perfusion with the NMDA receptor antagonist, 2-AP5 (30 μM, *n*=7) marginally reduced E_Bg_ (from 3.2±0.9 nS to 2.8±0.5 nS; *P*>0.05; Fig. 4A). At the same time, I_Bg_ fell from 15.0±5.6 nS to 10.1±3.0 nS (*P*>0.05). As noted above, NMDA receptors are unlikely to contribute directly to spontaneous excitation in entorhinal neurons. However, our previous patch clamp studies have shown that presynaptic NMDA receptors tonically facilitate glutamate release in the EC (Berretta and Jones, 1996b; Woodhall et al., 2001; Yang et al., 2006). Thus, the small reduction in E_Bg_ may well reflect an indirect effect resulting from decreased glutamate release and reduced activation of postsynaptic AMPA receptors resulting from blockade of the tonic facilitation mediated by presynaptic NMDA receptors. It is also the case that the excitatory drive onto EC interneurons has a powerful NMDA-receptor component (Jones and Buhl, 1993), so the reduction in I_Bg_ could result from blockade of NMDA receptors on the interneurons.

These possible explanations for the effects of 2-AP5 gain support from the observations that the effects of the drug appeared to be dependent on the baseline activity in control conditions. Thus, in four cells 2-AP5 appeared to have no, or minimal, effects on E_Bg_ or I_Bg_. However, in the remaining three cells baseline activity was high and clear effects were seen. In these “high-activity” cells, E_Bg_ fell from 5.4±1.8 to 1.7±0.6 nS in the presence of the antagonist, whereas I_Bg_ was reduced from 32.1±11.9 nS to 9.8±3.7 nS, and the I:E was unchanged (5.4±0.3 versus 5.6±1.1).

Overall, in all cells, the I:E increased slightly from 4.3±0.6 to 4.7±1.0 in favor of inhibition in the presence of 2-AP5. This was accompanied by a small increase in spike threshold from 20.4±1.2 mV to 23.4±1.6 mV (*P*=0.01). However, the number of spikes evoked by a supra-threshold train was not significantly affected (4.4±0.5 versus 3.5±0.7; Fig. 4A). Resting potential was unaltered by 2-AP5.

### Blockade of GluR5 kainate receptors

In other studies in this laboratory we have been studying the role of GluR5-containing kainate receptors in layer II of the EC. We have found that these receptors can enhance the release of both glutamate and GABA onto layer III cells, but neither effect is tonically activated by ambient glutamate, and that these receptors may make a contribution to the excitatory drive onto interneurons (Chamberlain and Jones, 2006; 2007). West et al. (2007) have recently reported that kainate receptors may partially mediate excitatory drive onto principal cells. We have also seen a contribution of kainate receptors to evoked EPSCs in about 50% of principal cells, although this is small contribution and unaffected by the selective GluR5 antagonist, UBP-302 (S. E. L. Chamberlain and R. S. G. Jones, unpublished observations). In the present study we looked at the effect of this antagonist on background conductances. UBP-302 (20 μM, *n*=6, Fig. 4B), had little effect on E_Bg_ (2.7±1.0 nS versus 2.2±1.2 nS), I_Bg_ (10.8±3.4 nS versus 10.3±4.9 nS) or the I:E (4.6±0.4 versus 4.8±0.4). This clearly indicates that under baseline conditions at least, GluR5 kainate receptors contribute little to the control of the background synaptic activity in layer III. Resting potential was also unaltered by UBP-302, although there did appear to be a small increase in spike threshold (from 18.2±1.3 mV to 21.5±1.4 mV). The number of spikes per train was largely unaltered (4.4±0.6 to 3.7±0.7). We cannot rule out a role of non-GluR5 containing kainate receptors to background synaptic activity, but currently available pharmacological tools do not allow us to specifically study the role of such receptors.

## Discussion

Sub-threshold background synaptic activity or “synaptic noise” is increasingly seen as a functional way of controlling excitability and gain in cortical neurons, and hence of the processing capabilities of cortical networks. Conversely, the characteristics of the background activity in individual elements provide information concerning the dynamic state of the network. Recording excitability of network elements and simultaneously quantifying the level of background activity presents considerable technical problems, and most studies have employed a dynamic clamp approach to model *in vivo*–like synaptic noise and determine its effects on gain and excitability. The V_m_D method devised by Rudolph et al. (2004) potentially allows us to quantify excitatory and inhibitory synaptic noise at the same time as monitoring excitability. In the present experiments we have shown that this method is a valid and useful approach for the investigation of network activity even under the quiescent conditions present in acute brain slices. It is clearly possible to realistically obtain a simultaneous quantification of the naturally arising background synaptic inhibition and excitation in entorhinal neurons in resting conditions, and to correlate changes in the ratio between the two to intrinsic excitability. Thus, we have provided further evidence to support the concept that background synaptic noise is an important determinant of neuronal excitability, and thus integration of activity in cortical networks.

Destexhe’s group (Rudolph et al., 2004) has reported a dominance of inhibition over excitation during spontaneous up states recorded from ferret visual cortex slices *in vitro*, and in cat parietal neurons under ketamine/xylazine anesthesia *in vivo*, using the V_m_D method (Rudolph et al., 2005). A similar ratio in favor of inhibition was recorded in the same animals during EEG-activated states following electrical stimulation in the pontine reticular formation. More recently, Destexhe’s group (Rudolph et al., 2007) has demonstrated dominant inhibition during natural sleep/waking states in non-anesthetized cats. Other studies using different approaches have previously suggested dominant inhibitory conductances in cat visual cortical neurons under anesthesia *in vivo* (Borg-Graham et al., 1998; Hirsch et al., 1998). However, it should be noted that others have provided evidence to suggest that excitatory and inhibitory conductances are approximately equal during spontaneous up states in ferret cortex *in vitro* or *in vivo* (Shu et al., 2003b; Haider et al., 2006). In contrast, the VmD method applied to neurons in the anesthetized (urethane) rat somatosensory cortex suggests that spontaneous up states are associated with a rise in excitatory conductance with a concurrent fall in inhibition (Zou et al., 2005). Our data add to these observations by showing that under quiescent resting conditions in layer III of the EC *in vitro*, background synaptic noise mediated by GABA strongly dominates over excitation. This concurs with our whole cell patch clamp experiments, which have shown that under the same conditions, sIPSCs are larger and more frequent than sEPSCs (see above).

We have previously shown that neurons in the superficial layers of the EC display spontaneous up states under various conditions (Jones and Heinemann, 1989; Cunningham et al., 2006). The synaptic balance at rest demonstrated suggests an inherently stable low-excitation–high-inhibition network, and we now plan to use the V_m_D approach to determine the dynamic relationships between E_Bg_ and I_Bg_ during the switch from quiescent to activated states. It is too early to say what the scaling of the I:E may be leading to and during activation. It is possible that the relationship may be linearly scaled, tend toward an equal balance or even reverse (cf. Shu et al., 2003; Zou et al., 2005; Rudolph et al., 2007). However, it is clear from our pharmacological data that network balance in the EC is a dynamic one even at apparent rest. For example, blocking glutamate receptors not only depresses synaptic excitation, but by reducing activity driven drive onto the interneurons, will simultaneously lead to a decrement in feed-forward/feedback inhibition. However, the overall change was to tilt the balance more strongly in favor of inhibition, and presumably a further stabilization of the network. In contrast, loss of inhibition in the presence of bicuculline resulted in only a slight increase in excitation, but a dramatic decrease in I:E in the lead up to epileptiform discharges. Generally, all the pharmacological manipulations we employed induced changes in E_Bg_ and I_Bg_ that were explicable in terms of static or dynamic network changes, giving us confidence that the V_m_D method is a valuable tool to investigate changes in entorhinal network activity.

It was also apparent that changes in background synaptic activity could be accompanied by changes in excitability in principal neurons. In many cases, these changes logically reflected changes in the I:E, and when this favored excitation then excitability increased, with the opposite situation arising when inhibition became more dominant. While we cannot directly link the perturbations in excitability to the changes in background synaptic activity, it seems highly likely that they are related, and that cortical processing would be altered across the network as a result of manipulation of background activity. It is clear from a number of investigations that changes in E_Bg_ and/or I_Bg_ can alter neuronal responsiveness in terms of firing output elicited by excitatory drive. For example, a number of studies have shown that increasing background synaptic noise has a divisive action and reduces the slope of the input–output relationship thereby modulating gain (e.g. Chance et al., 2002; Mitchell and Silver, 2003). The importance of the functional effects of such a gain change is illustrated by the ability of background synaptic noise fluctuations to actively control thalamo-cortical transfer function (Wolfart et al., 2005). The ability to realistically quantify E_Bg_ and I_Bg_ demonstrated here opens the way for us to determine how gain is altered during changing network conditions in EC neurons, and allows us to examine how such changes influence EC-hippocampal transfer.

Finally, the EC is a pivotal site in the generation of temporal lobe epilepsies (see Jones and Woodhall, 2005). We plan to use the V_m_D approach to examine changes in synaptic organization during chronic epilepsy, to determine if changes in background synaptic activity result in altered network stability and excitability leading to epileptogenesis. Furthermore, we will study how anticonvulsant drugs alter background activity and consequent excitability. It is clearly the case that these (indeed any centrally acting) drugs have access to all elements of cortical networks after systemic administration, and examination of dynamic changes induced by the drugs will provide important information concerning their overall therapeutic actions.
